# Antimicrobial Activity of *Stryphnodendron adstringens* (Mart.) Coville, *Baccharis crispa* Spreng, and *Azadirachta indica* Against Bacteria Causing Bovine Mastitis and Phytochemical Profiling Determined by PS-MS

**DOI:** 10.3390/cimb48060573

**Published:** 2026-05-29

**Authors:** Gian Carlos Nascimento, Melina Laura Moretti Pinheiro, Brenda Veridiane Dias, Raphael Ocelli Pinheiro, Maria Aparecida Vasconcelos Paiva Brito, Afonso Henrique de Oliveira Júnior, Lara Louzada Aguiar, Rodinei Augusti, Julio Onesio-Ferreira Melo, Rafael Bastos Teixeira, Ana Cardoso Clemente Filha Ferreira de Paula

**Affiliations:** 1 Department of Agricultural Sciences, Federal Institute of Minas Gerais Campus Bambuí, Fazenda Varginha–Rodovia Bambuí/Medeiros–Km 05, Bambuí 38900-000, MG, Brazil; melinalaura@yahoo.com.br (M.L.M.P.); brendavdias@hotmail.com (B.V.D.); rafael.teixeira@ifmg.edu.br (R.B.T.); ana.paula@ifmg.edu.br (A.C.C.F.F.d.P.); 2 Innovation Agency (NIT/AI-RE), Federal Institute of Minas Gerais, Avenida Professor Mário Werneck, 2590, Belo Horizonte 30575-180, MG, Brazil; raphaeloce@hotmail.com; 3 The Brazilian Agricultural Research Cooperation (EMBRAPA), Brasília 70770-901, DF, Brazil; mavpaiva@gmail.com; 4 Department of Exact and Biological Sciences, Sete Lagoas Campus, Federal University of São João del-Rei, Sete Lagoas 36307-352, MG, Brazil; afonsohoj@gmail.com (A.H.d.O.J.); laralaguiar@hotmail.com (L.L.A.); 5 Department of Chemistry, Belo Horizonte Campus, Federal University of Minas Gerais, Belo Horizonte 31270-901, MG, Brazil; augusti.rodinei@gmail.com; 6 LEAF Superior Institute of Agronomy, University of Lisbon, 1649-004 Lisbon, Portugal

**Keywords:** MBC, plant extracts, antimicrobial activity, Phytotherapy, paper spray ionization, mass spectrometry, barbatimão, carqueja, neem

## Abstract

Medicinal plants have attracted increasing scientific interest due to the diversity of bioactive compounds reported across different species. They may represent complementary sources of bioactive compounds alongside conventional antimicrobials, which may pose risks to animal health and compromise treatment efficacy. Considering the importance of alternative compounds, we aimed to evaluate the antimicrobial activity in vitro of medicinal plants *Stryphnodendron adstringens* (Mart.) Coville, known as barbatimão, *Baccharis crispa* Spreng, known as carqueja and *Azadirachta indica*, known as neem. *S. adstringens* (Mart.) Coville and *B. crispa* Spreng were used as extract and obtained from plants collected in the municipality of Bambuí, state of Minas Gerais, Brazil. *A. indica* was evaluated as extract and oil, and the crushed leaves and oil were purchased from a commercial company. Antimicrobial activity was determined by the minimum bactericidal concentration (MBC) test-against *Staphylococcus aureus*, *Streptococcus agalactiae*, *Streptococcus uberis*, *Escherichia coli*, and *Salmonella* spp., isolated from bovine mastitis. The bacteria were submitted to the MBC test at concentrations of 100, 50, 25, 12.5, 6.25, 3.12, 1.56, 0.78, 0.39, 0.19 and 0.09 mg/mL. The bacteria evaluated were sensitive to most plant extracts for at least one of the concentrations evaluated, except for Gram-negative bacteria, *Escherichia coli*, and *Salmonella* spp. There was no activity of *B. crispa* Spreng extract and *A. indica* against *E. coli* and neither of *B. crispa* Spreng extract against *Salmonella* spp. even at the highest concentration evaluated. *S. adstringens* (Mart.) Coville was considered the extract with the highest activity against the bacteria evaluated and *S. uberis* the most susceptible to antimicrobial action. The results indicate detectable antimicrobial activity of the evaluated extracts and oil, suggesting their potential relevance as complementary sources of bioactive compounds for further investigation, rather than as direct alternatives to conventional antibiotic therapies. Paper spray mass spectrometry (PS-MS) was employed as an exploratory phytochemical screening approach, and all metabolite assignments reported herein should be regarded as tentative or putative annotations under the analytical conditions used, consistent with MSI Level 3 confidence.

## 1. Introduction

Animal health has a major impact on the quality of the final product in the milk production chain. In this sense, bovine mastitis is highly relevant, as it is the primary infection in dairy cattle, usually caused by bacteria, especially those of the genera Staphylococcus and Streptococcus [1]. In addition to representing a global problem, scientific reports show an increase in mastitis cases, partly due to the selection of animals for higher milk production and the use of broad-spectrum antibiotics [2,3,4]. Due to the high prevalence and the significant losses it causes, mastitis is considered the costliest disease in dairy production [5].

The emergence and spread of antimicrobial resistance to commonly used antibiotics have increased the demand for alternative or complementary antimicrobial strategies that may support existing treatment approaches [6]. In addition to this necessity, there has been a growing interest in medicinal plants as sources of new bioactive molecules against bacteria [6,7]. The bioactivity of medicinal plants is inherent to a group of compounds called secondary metabolites. These compounds are synthesized through catabolic, anabolic, and biotransformation reactions involving amino acids, carbohydrates, and lipids produced by the plant. Several crude extracts containing antimicrobial metabolites that inhibit bacterial growth have been reported [6,7].

*Stryphnodendron adstringens* (Mart.) Coville, popularly known as barbatimão, stands out as one of the most widely used plants in medicinal treatments, particularly because of the tannins in its bark, which have been associated with biological properties, including antimicrobial activity against *Staphylococcus aureus*, *Pseudomonas* spp., and *Escherichia coli* [8,9,10]. *Baccharis crispa* Spreng, known by the name of carqueja, is popularly used as an antiseptic [11] and as an antioxidant [12]. *Azadirachta indica*, known as neem, has been widely investigated for insecticidal, fungicidal, nematocidal, and antibacterial-related properties, including insecticidal [13] and fungicidal activity, nematode control [14], and antibacterial activity [15].

Considering the potential of these medicinal plants, we aimed to determine, using the minimum bactericidal concentration (MBC), the in vitro antimicrobial effect of plant extracts of *Stryphnodendron adstringens* (Mart.) Coville, *Baccharis crispa* Spreng, and *Azadirachta indica* and of *Azadirachta indica* oil against strains of *Staphylococcus aureus*, *Streptococcus agalactiae*, *Streptococcus uberis*, *Escherichia coli*, and *Salmonella* spp. isolated from bovine mastitis. A complementary objective was to tentatively identify the secondary metabolites present in the evaluated extracts using the ambient ionization methodology for Paper Spray Mass Spectrometry (PS-MS). Once antimicrobial potential is detected it is possible to investigate alternatives to the use of antimicrobials against the main bacteria that cause bovine mastitis. Although PS-MS has been previously employed for rapid phytochemical profiling of medicinal plants, its previous use does not include the species from this study (i.e., *Stryphnodendron adstringens* (Mart.) Coville, *Baccharis crispa* Spreng, and *Azadirachta indica*), it has largely remained descriptive and disconnected from biological outcome modeling. In this study, PS-MS is employed alongside minimum bactericidal concentration assays and generalized linear modeling (GLM) as a complementary exploratory tool to obtain a rapid phytochemical fingerprint of the evaluated plant-derived matrices. Rather than providing mechanistic or functional evidence, the PS-MS data are used descriptively to characterize broad chemical classes present in each preparation, offering a qualitative context for the antimicrobial findings without implying causal attribution to specific compounds.

Paper Spray Mass Spectrometry was incorporated into this study as a rapid ambient ionization strategy to generate an exploratory qualitative fingerprint of the evaluated plant-derived matrices, and not as a platform for structural elucidation or mechanistic inference. The phytochemical profiles obtained are intended solely to describe the broad chemical classes present in each preparation under the analytical conditions employed, providing descriptive context for the antimicrobial findings. No causal relationship between detected signals and bactericidal outcomes is implied or supported by this approach. The value of PS-MS in this context lies in its ability to rapidly compare the general phytochemical composition of chemically distinct matrices with minimal sample preparation, which may inform the design of future targeted investigations.

## 2. Materials and Methods

### 2.1. Material Sampling and Extract Preparation

Plants *S. adstringens* (Mart.) Coville (−20.041632, −46.022234) and *B. crispa* Spreng (−20.040778, −46.021444) were collected in the municipality of Bambuí, state of Minas Gerais in Brazil, and then deposited in the Herbarium of the Agricultural Research Company of Minas Gerais (EPAMIG) (Belo Horizonte, MG, Brazil), registry 58172 and 58173, respectively. The crushed leaves of *A. indica* were provided by the company GoNeem (Los Angeles, CA, USA), and *A. indica* oil (OAi) extracted from the plant seeds by cold maceration, was obtained from the company Globo Agronegócios e Participações Ltd. (Ponta Pora, MS, Brazil).

The extract of *S. adstringens* (Mart.) Coville (ESa) was obtained by maceration, from 428.57 g of dry matter (DM) of the ground bark, in one liter of absolute ethyl alcohol PA for eight days, and the solvent was renewed every four days [16,17]. The extract of *B. crispa* Spreng (EBc) was prepared from the aerial part of the dry plant at room temperature and obtained by decoction for 15 min from 25 g of DM of the ground plant for each liter of distilled water [18,19]. The extract of *A. indica* (EAi) was obtained from the maceration of 100 g of DM of the crushed leaves in one liter of absolute ethyl alcohol PA for eight days, with the renewal of the solvent every four days [20,21]. All extracts were vacuum-filtered using paper filters (80 g/m^2^).

ESa and EAi were concentrated using roto-evaporator *Fisatom* 801 (São Paulo, SP, Brazil) at 45 °C. The semi-solid residue was placed in an oven at a temperature of 45 °C for seven days for total drying. The EBc was kept in a freezer at −80 °C and then freeze-dried using the L101 Liotop^®^ (São Carlos, SP, Brazil). The extracts and OAi were used to evaluate the bactericidal activity after the sterility certification proposed by the Brazilian Pharmacopoeia (2010) [22].

### 2.2. Strains

Five strains isolated from bovine mastitis cases were used. They belong to the Collection of Microorganisms of Interest of Agroindustry and Livestock (CMIAP) of Embrapa Dairy Cattle. The following bacterial species were evaluated: *Staphylococcus aureus*, *Streptococcus agalactiae*, *Streptococcus uberis*, *Escherichia coli*, and *Salmonella* spp.

### 2.3. Inoculum Preparation

The bacterial strains *Staphylococcus aureus* (strain no. 35), *Streptococcus agalactiae* (strain no. 39), *Streptococcus uberis* (strain no. 13), *Escherichia coli* (strain no. 3890), and *Salmonella* sp. (strain no. 5255), isolated from cases of bovine mastitis, were obtained from the Collection of Microorganisms of Interest to Agribusiness and Livestock (CMIAP) of Embrapa Dairy Cattle (Juiz de Fora, Minas Gerais, Brazil). This institutional collection comprises bacterial lineages previously isolated from bovine milk samples associated with mastitis and preserved for scientific research purposes. The selected strains represent the most relevant mastitis pathogens, covering both contagious and environmental profiles.

The strains were maintained as stock cultures in skim milk (Difco^®^) (Franklin Lakes, NJ, USA) supplemented with 10% (*v*/*v*) glycerol and stored at −80 °C for long-term preservation, while working aliquots were kept at −20 °C, in accordance with the collection’s maintenance protocols. For reactivation and subsequent use, an aliquot of the frozen bacterial suspension was streaked onto Petri dishes containing Ivantone Soya Agar (ISA) (Difco^®^) supplemented with 5% (*v*/*v*) defibrinated sheep blood. The plates were incubated at 35 °C for 24 h.

All procedures were performed under aseptic conditions to ensure cell viability, phenotypic stability, and experimental reproducibility. Strain identification and traceability were ensured through the internal CMIAP collection codes, as described by Brito (Collection of Microorganisms of Interest to Agribusiness and Livestock–Embrapa Dairy Cattle; available at: https://www.infoteca.cnptia.embrapa.br/infoteca/bitstream/doc/1128717/1/Banco-germoplasma.pdf; accessed on 8 January 2016).

The standardization of bacterial suspensions was performed according to the method proposed by the Clinical and Laboratory Standards Institute [23]. The inoculum was prepared by suspending from four to five colonies previously grown in Brain Heart Infusion (BHI, Difco^®^) in a test tube containing Mueller-Hinton broth (MH, Kasvi^®^) (Weissópolis Pinhais, PR, Brazil). The turbidity was adjusted according to the standard of 0.5 of the McFarland scale by visual comparison, corresponding to approximately 1.5 × 10^8^ CFU/mL. The inoculums were standardized at the concentration of 1 to 3 × 10^6^ CFU/mL for all bacteria and then dispensed in each test tube. MH broth was used as a culture medium for MBC tests. For the bacteria *S. agalactiae* and *S. uberis*, 5% sterile defibrinated equine serum was added.

### 2.4. Determination of Minimum Bactericidal Concentration (MBC)

The MBCs of the extracts and the OAi were determined by the broth macro dilution method as proposed by CLSI (2008). One mL of the extracts and OAi reconstituted in aqueous solution containing DMSO and Tween 80 [24] at the concentration of 200 mg/mL was diluted twice in MH broth to obtain concentrations 100, 50, 25, 12.5, 6.25, 3.12, 1.56 and 0.78, 0.39, 0.19 and 0.09 mg/mL for each extract and OAi.

The solution containing DMSO and TWEEN 80 was used as a negative control, and antibiotics oxacillin or neomycin were used as a positive control, the first for Gram-positive bacteria and the second for Gram-negative bacteria. It should be noted that DMSO and TWEEN 80, even at low concentrations, may exert membrane-active effects on bacterial cells. To minimize this potential confounding factor, the vehicle was maintained at the same final concentration across all dilutions and tested independently as the negative control, confirming the absence of bactericidal activity attributable to the solvent system alone under the conditions evaluated. Positive controls were used to validate assay performance rather than to establish comparative efficacy. One mL of the bacterial inoculum standardized in 1 to 3 × 10^6^ CFU/mL was inoculated in each of the test tubes. Then, the test tubes were incubated at 35 °C ± 2 °C for 24 h. Subsequently, an aliquot of 10 μL of each dilution was spread out onto Petri dishes containing BHI agar (Difco^®^) and incubated at 35 °C ± 2 °C for 18–24 h. The MBC was considered as the lowest concentration of extracts that did not present bacterial growth. The tests to evaluate the bactericidal activity of the extracts and oil were executed in triplicates.

Due to the intrinsic physicochemical properties of the crude plant extracts, particularly their intense coloration and optical interference, conventional turbidity-based methods for MIC determination were deemed unreliable. Both visual assessment of bacterial growth and spectrophotometric measurements were significantly compromised by background absorbance associated with the extracts. In the absence of alternative viability indicators (e.g., resazurin-based assays), MIC values could not be accurately established.

Consequently, bactericidal activity was evaluated through the determination of the MBC, performed by subculturing aliquots from all tested concentrations onto agar plates, followed by incubation to assess bacterial viability. This approach enabled the identification of the lowest concentration capable of completely preventing bacterial regrowth.

Although MIC is a widely used reference endpoint in antimicrobial susceptibility testing, the present study focused exclusively on MBC to characterize bactericidal—rather than bacteriostatic—activity, which is more relevant in the context of mastitis pathogens that must be eliminated rather than merely suppressed in an intramammary environment. The choice of MBC as the primary endpoint is consistent with studies aimed at evaluating the therapeutic ceiling of plant-derived preparations, where confirming the absence of viable cells is methodologically more informative than growth inhibition alone.

It is acknowledged that this approach departs from the conventional sequential CLSI workflow in which MBC is derived from the MIC wells showing no visible growth, and that the absence of MIC data prevents calculation of the MBC/MIC ratio. Consequently, the formal distinction between bactericidal and bacteriostatic effects at sub-MBC concentrations cannot be established from the present dataset and represents a recognized limitation of the study design.

### 2.5. Paper Spray Mass Spectrometry

A sample of 1 g of each extract (ESa, EBc and OAi) was weighed separately on an analytical balance and added to 8 mL of methanol (HPLC grade) in a Falcon flask. These mixtures were vortexed for 30 s and left at rest for one hour at room temperature.

The chemical profile of the extracts was determined according to the methodology described by Ramos et al. [18] using an LCQ Fleet mass spectrometer (ThermoScientific^®^) (Waltham, MA, USA) coupled with an ionization source for paper pulverizing. For this, chromatographic paper in the shape of an equilateral triangle (1.5 cm) was used, placed at a distance of 0.5 cm from the spectrometer entrance in a metal connector. 2 μL of the extract, and 40 μL of methanol was applied to the paper, reading in triplicate in positive and negative ionization modes.

The instrumental conditions of the analyses were: source voltage at 4.5 kV for the positive mode and 3.5 kV for the negative mode; capillary voltage of 40 V; transfer tube temperature of 275 °C; tube lens voltage of 120 V; mass range from 100 to 1000 *m*/*z*. Metabolite annotations were obtained by comparing nominal *m*/*z* values from literature reports with the instrumental signals observed and, where available, by inspection of MS/MS data acquired at collision energies of 15–30 au (arbitrary units) as supportive information only. Given the use of a low-resolution ion trap instrument and the complexity of the plant matrices analyzed, these assignments were treated conservatively as tentative/putative annotations rather than definitive compound identifications. According to the level of analytical confidence supported by the present workflow, the reported annotations should be interpreted as corresponding to MSI Level 3.

The analytical purpose of PS-MS in the present study was to obtain comparative phytochemical fingerprints capable of supporting the biological interpretation of the MBC assays. Because PS-MS enables direct ionization of complex extracts with minimal sample preparation, it is particularly suitable for exploratory screening of crude medicinal plant matrices, where the objective is to detect dominant ion signals and infer broad chemical classes rather than to quantify individual compounds. Accordingly, the spectra were used to guide discussion of the antimicrobial outcomes at the extract and oil level, with emphasis on putative tannins, flavonoids, phenolic acids, proanthocyanidins, and lipophilic constituents. This approach preserves analytical caution while still allowing PS-MS data to contribute relevant context to the antimicrobial findings, especially when differences in bactericidal performance were observed among extracts prepared from different plant species or matrices.

Therefore, PS-MS was used in this study as an exploratory qualitative profiling tool and not as a platform for confident molecular formula assignment or structural elucidation.

### 2.6. Statistical Analysis

The statistical analysis was performed with the help of the statistical program R (Version 4.5.1, 2025) [25]. For the analysis of the bactericidal action of the extracts and the OAi, the generalized linear model (GLM) of the MASS package was used, with the response modeled by Bernoulli distribution and logit link function to evaluate the effects of extract type, concentration, and bacterial species on the probability of bactericidal response, allowing comparisons among treatments while accounting for the binary nature of the outcome (presence/absence).

The analysis of deviance (ANODEV) was used in a completely randomized design. Value 0 was assigned when there were bactericidal action and value 1 when there was bacterial growth. The significance level was established in *p* < 0.01. For graphical analysis, the ggplot function of the ggplot2 package [25] was used. CBM response variable is the following:

Y_i_~Bernoulli (μ_i_)

Y_i_: when there was bacterial growth (1) and when there was bactericidal action (0).

μ_i_: Bernoulli distribution with logit link function.

To reduce potential biases associated with complete data separation, the models were fitted using bias-reduced maximum likelihood estimation, as implemented in the brglm2 package. Adjusted means were obtained using the emmeans package and compared through multiple comparison tests with Sidak adjustment (α = 0.01). Lowercase letters indicate comparisons among extracts within each concentration for a given bacterial species, whereas uppercase letters denote comparisons among concentrations within each extract. Predicted probabilities were expressed as percentages and are presented with their respective confidence intervals.

## 3. Results and Discussion

### 3.1. Antimicrobial Activity

Bactericidal activity was observed at concentrations ranging from 1.56 to 100 (mg/mL). However, due to the intrinsic optical interference of the crude extracts, MIC values could not be reliably determined. Consequently, although bactericidal effects were clearly identified at the tested concentrations, no classification based on the MBC/MIC ratio could be established. All evaluated extracts exhibited detectable antimicrobial activity against at least one of the tested bacterial strains.

The ANODEV revealed a statistically significant interaction between extract type and concentration, indicating that bactericidal effects varied depending on the extract evaluated and the concentration applied (*p* < 0.01). However, differences regarding the concentrations used and regarding the species in question were observed. These concentrations highlight the exploratory nature of crude extracts and reinforce their relevance as screening matrices rather than as direct therapeutic agents.

**Figure 1:**
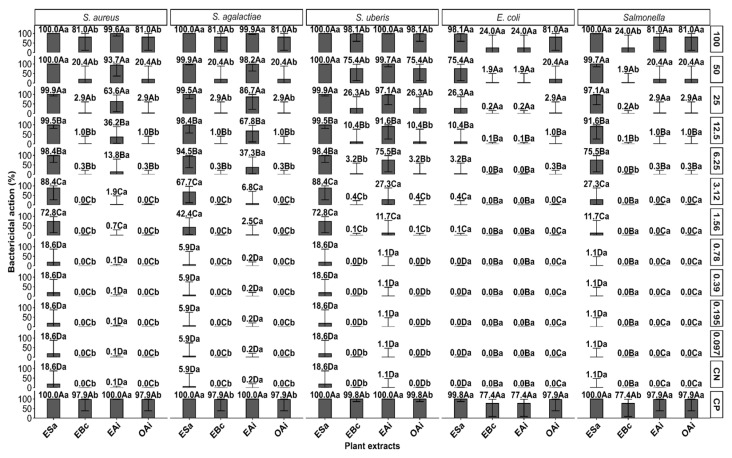
Bactericidal action (%) of plant extracts and oil against bovine mastitis–associated bacterial strains across decreasing concentrations. The bactericidal response (%) of *Staphylococcus aureus*, *Streptococcus agalactiae*, *Streptococcus uberis*, *Escherichia coli*, and *Salmonella* (i.e., *Salmonella* spp.) is shown for *Stryphnodendron adstringens* (Mart.) Coville extract (ESa), *Baccharis crispa* Spreng extract (EBc), *Azadirachta indica* extract (EAi), and *Azadirachta indica* oil (OAi) at concentrations ranging from 100 to 0.097 mg/mL. Legend: Values represent mean ± standard deviation of triplicate assays. Different uppercase letters indicate significant differences among concentrations within the same extract and bacterial species, while different lowercase letters indicate significant differences among extracts at the same concentration (GLM with Bernoulli distribution and ANODEV, *p* < 0.01). CN: negative control (DMSO/Tween 80); CP: positive control (oxacillin for Gram-positive bacteria and neomycin for Gram-negative bacteria).

To further visualize the interaction between extract type, concentration, and bacterial species identified by the GLM analysis, bactericidal responses were plotted across the full concentration range evaluated (Figure 1). This representation highlights the dose–response behavior of each extract and reveals marked differences in susceptibility among bacterial species that are not fully captured by discrete MBC values alone. In particular, Gram-positive bacteria exhibited a progressive decline in viability with decreasing concentrations, whereas Gram-negative species displayed an abrupt loss of bactericidal response below higher concentration thresholds. The figure also emphasizes matrix-dependent effects, with *S. adstringens* (Mart.) Coville extract showing sustained activity across a broader concentration range compared with *Baccharis crispa* Spreng and *Azadirachta indica* preparations.

The ESa showed an inhibitory effect against all bacterial species tested, but at different concentrations. The inhibitory effect against *E. coli* was observed only at high concentrations, with bactericidal activity detected at 50–100 mg/mL. A similar pattern was observed for OAi, which exhibited bactericidal activity against all tested species, albeit only at relatively higher concentrations than those used when the test was performed with ESa. For OAi, the lowest dose considered efficient was 50 mg/mL against *S. uberis*. Against other species, OAi was only able to inhibit bacterial growth when used at maximum concentration (100 mg/mL). EBc and EAi, even when used at the maximum concentration, could not inhibit *E. coli* growth, and considering the *Salmonella* spp., the same was observed for the maximum concentration of EBc (Table 1). Based on MBC values, *S. aureus* and *S. uberis* exhibited higher susceptibility to the evaluated extracts, while the extract of *S. adstringens* (Mart.) Coville showed comparatively lower MBC values under the experimental conditions; on the other hand, the least susceptible species was *E. coli*, which exhibited bactericidal response only when exposed to ESa and OAi at high concentrations (*p* < 0.001).

In addition to discrete MBC values, the concentration-resolved profiles revealed distinct bactericidal response patterns among bacterial groups and extracts (Figure 1). Gram-positive bacteria exhibited a progressive, concentration-dependent reduction in viability, with detectable bactericidal activity persisting across multiple dilution steps, particularly for *S. adstringens* (Mart.) Coville extract. In contrast, Gram-negative bacteria displayed an abrupt transition from high bactericidal response at elevated concentrations to near-complete loss of activity at lower doses, indicating a narrow effective concentration window. Across all extracts, *Streptococcus uberis* consistently showed higher susceptibility, followed by *Staphylococcus aureus* and *Streptococcus agalactiae*, whereas *Escherichia coli* and *Salmonella* spp. were markedly less responsive. Differences in response stability were also observed among extracts, with ESa maintaining activity over a broader concentration range compared with EBc, EAi, and OAi.

**Figure 2:**
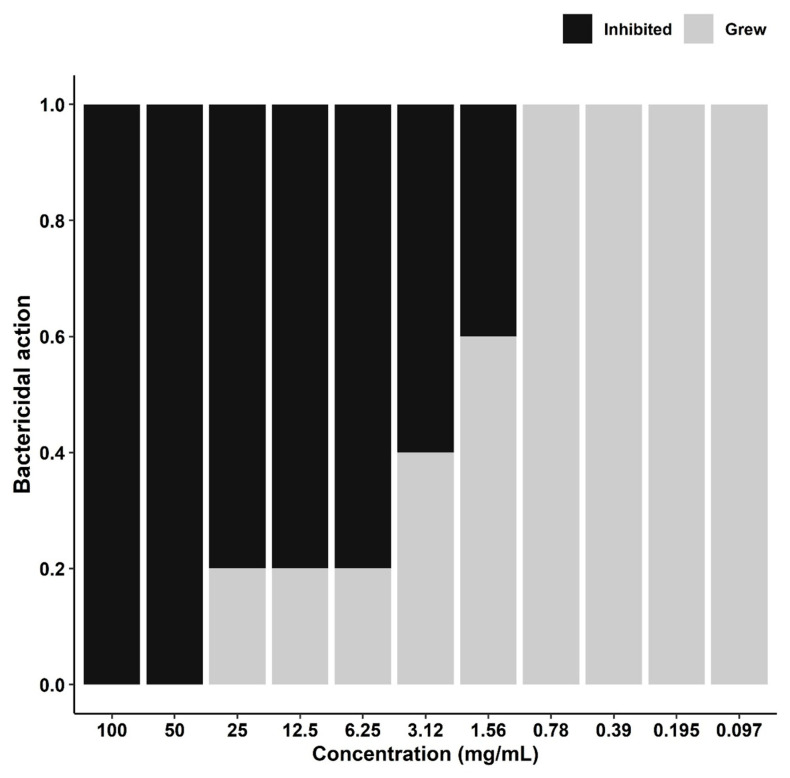
Bactericidal action of the different concentrations of *Strypnodendron adstringens* (Mart.) Coville against *S. aureus*, *S. agalactiae*, *S. uberis*, *E. coli*, and *Salmonella* spp.

When used at concentrations 0.097, 0.195, 0.39, and 0.78 mg/mL, ESa did not exhibit detectable bactericidal activity. As the concentration was increased, the bactericidal effect of ESa increased, showing different susceptibility of the bacteria studied to this extract. When used at a concentration of 50 mg/mL, ESa was effective against all species studied (Figure 2). Among the bacteria studied, *E. coli* was the least susceptible (growth inhibited at a concentration of 50 mg/mL), while *S. aureus* and *S. uberis* were the most susceptible to this extract (growth inhibited at concentrations of 1.56 mg/mL (Table 1).

Costa et al. [26] evaluated higher concentrations of ESa against bacteria isolated from milk samples, reaching 400 mg/mL, and classified *E. coli* as not susceptible to the extract since no bactericidal action was observed even at the highest concentration evaluated. The results obtained by these researchers differ from those observed in our study and showed the difference in susceptibility of microorganisms isolated from different sources, especially the environmental pathogen, which may originate from a variety of sources, including cattle bedding, manure, pastures and water [27].

Despite limiting the growth of both Gram-positive and Gram-negative species, lower ESa concentrations have more inhibitory activity in the growth of Gram-positive species, indicating the greater susceptibility of this group to the extract. The difference in sensitivity between Gram-positive and Gram-negative has been discussed previously and may be related to structural differences between these microorganisms. Gram-negative bacteria have an external phospholipid membrane with lipopolysaccharides, which makes the cell wall more complex and resistant to certain antimicrobial components. They are different from Gram-positive bacteria, which possess a thicker peptidoglycan layer, but lack the outer lipopolysaccharide membrane, which can result in higher permeability to certain antimicrobial compounds [26,28,29].

**Figure 3:**
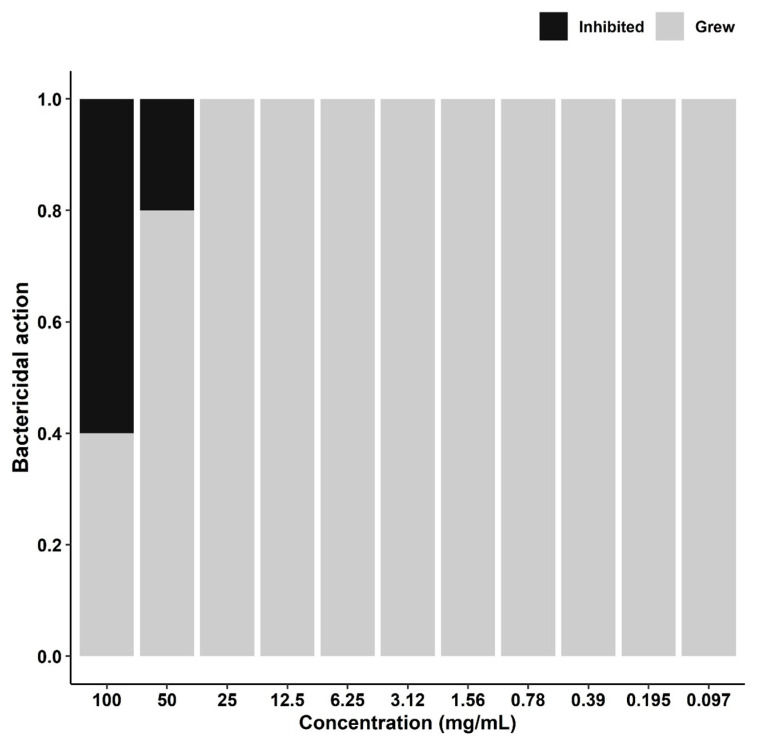
Bactericidal action among the different concentrations of *Baccharis crispa* Spreng against *S. aureus*, *S. agalactiae*, *S. uberis*, *E. coli*, and *Salmonella* spp.

Unlike ESa, EBc did not exhibit significant antibacterial activity against the microorganisms tested. It was not possible to find the MBC for *E. coli* and *Salmonella* spp. (Table 1). For these two species, MBC was higher than 100 mg/mL and was considered not susceptible to the extract. Since EBc did not present bactericidal action against Gram-negative bacteria due to the lower susceptibility of these bacteria, the difference (*p* < 0.001) was observed in the concentrations evaluated against *S. aureus*, *S. agalactiae*, and *S. uberis*. EBc at the concentration of 100 mg/mL presented higher bactericidal potential when compared with the use at the concentration of 50 mg/mL, which in turn was effective only against *S. uberis* (Figure 3).

Palacios et al. [30] evaluated the antimicrobial power of *B. crispa* Spreng oil by the presence of a disk diffusion test and found the antimicrobial potential against *S. aureus.* They also did not find bactericidal action against *E. coli*. These authors were the first to describe the antimicrobial potential of *B. crispa* Spreng and concluded that this potential is partial and variable by the action of the compounds present according to the species.

The growth inhibition observed for *S. uberis*, *S. aureus*, and *S. agalactiae* at higher concentrations of EBc is noteworthy, although its activity remained limited and concentration dependent. The species *S. aureus*, *S. uberis*, *S. agalactiae*, and coliforms can represent up to 80% of isolated pathogens [31]. Considering the importance of inhibiting these pathogens, Avancini et al. [19] evaluated the bactericidal power of *Baccharis trimera* and suggested the possibility of using plant extracts as a natural disinfectant and biological antiseptic in certain problem situations in animal production, specifically related to agents *S. aureus* and *S. uberis*.

The medicinal efficacy of the Indian plant *Azadirachta indica*, popularly known as neem has been the target of several studies, and the biologically active extracts obtained from its leaves, fruits, seeds, and trunk are recognized by their multiple therapeutic properties, insecticides, nematicides, and fungicides [32,33]. In this study, the bactericidal power of the plant was evaluated in oil (OAi) and extract (EAi).

**Figure 4:**
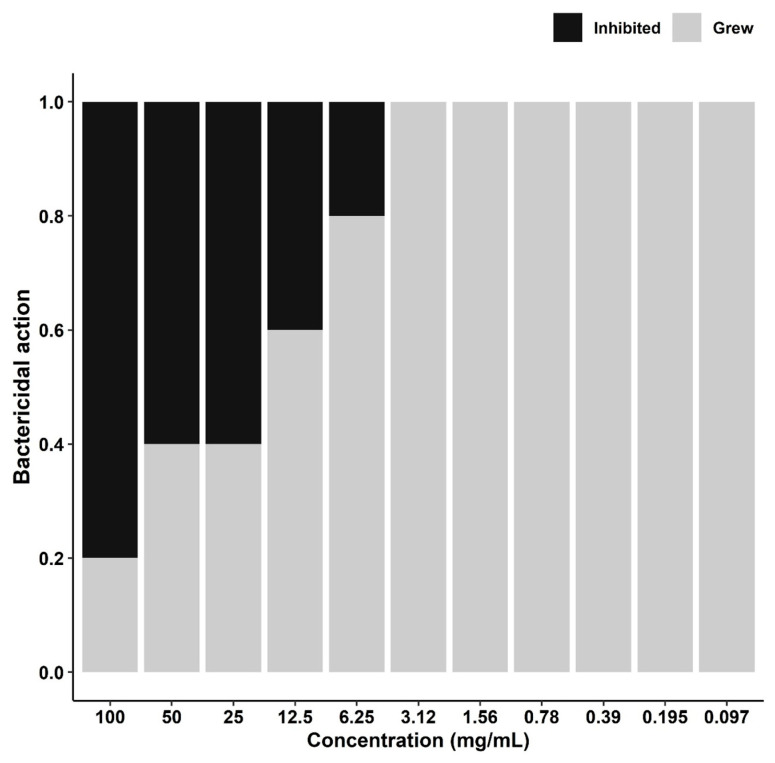
Bactericidal action between the different concentrations of oil *Azadirachta indica* in front of *S. aureus*, *S. agalactiae*, *S. uberis*, *E. coli*, and *Salmonella* spp.

The bactericidal action of the EAi was detected for most of the species studied, except for *E. coli*, for which MBC was not determined (Table 1). The concentrations of EAi used promoted different bactericidal efficacy according to the bacteria (*p* < 0.01). At a concentration of 100 mg/mL, the bactericidal action was effective against *S. aureus*, *S. agalactiae*, *S. uberis*, and *Salmonella* spp. As observed when in contact with EBc, the most susceptible pathogen was *S. uberis*, being inhibited with 50 mg/mL. When used at a concentration of 3.12 mg/mL or lower, EAi was not efficient against any of the species evaluated (Figure 4).

**Figure 5:**
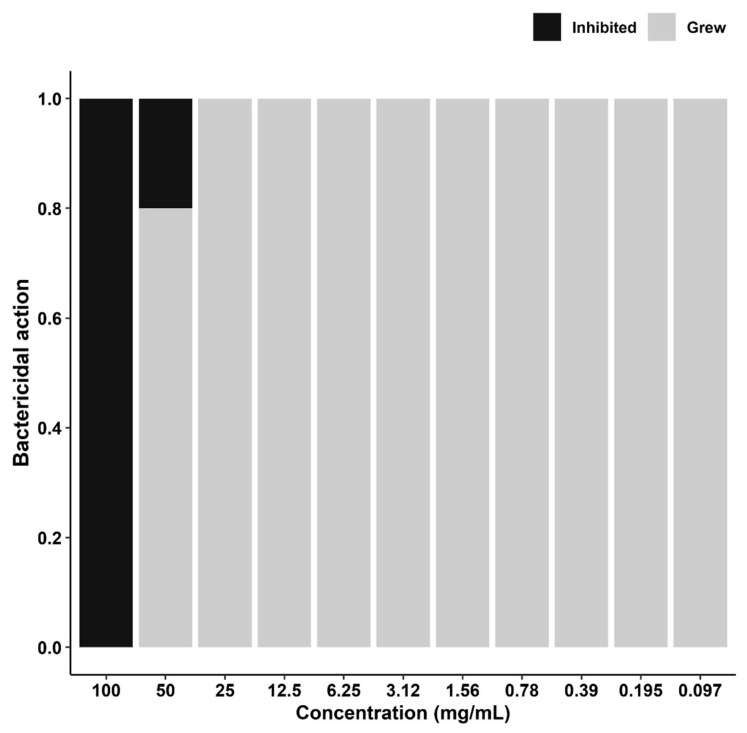
Bactericidal action of different concentrations of *Azadirachta indica* oil against *S. aureus*, *S. agalactiae*, *S. uberis*, *E. coli*, and *Salmonella* spp.

The *A. indica* oil showed lower MBC values since lower concentrations were used to inhibit bacterial growth (Table 1). OAi showed satisfactory antibacterial activity only if used at the maximum concentration (100 mg/mL) for most bacteria, except for *S. uberis*, in which MBC was 50 mg/mL (*p* < 0.001, Figure 5).

Although EAi is generally considered more efficient, we found that OAi exhibited bactericidal activity at lower concentrations against *E. coli* than EAi since *E. coli* was considered not susceptible to the extract. This indicates that oil compounds can be more efficient in certain situations. Arroteia et al. [33] evaluated the effect of *A. indica* on the inhibition of a mycotoxin production found in apples and reported a higher potential of *A. indica* in oil, indicating that liposolubility would be an activating potential of its activity. As reported here, the action of oil is not always more efficient, and besides the scope of action against microorganisms, production costs should be considered. The use of plant extracts may be economically more feasible than oils, as oil extraction generally requires greater processing and resource input [34]. The findings should be interpreted strictly within the context of in vitro screening.

### 3.2. Exploratory Phytochemical Profiling by Paper Spray Mass Spectrometry (PS-MS)

**Figure 6:**
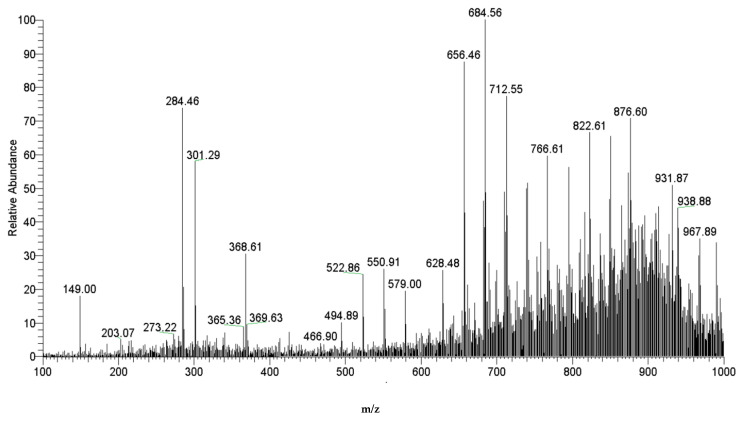
Representative full-scan PS-MS profile of *Stryphnodendron adstringens* (Mart.) Coville extract in positive ionization mode.

**Figure 7:**
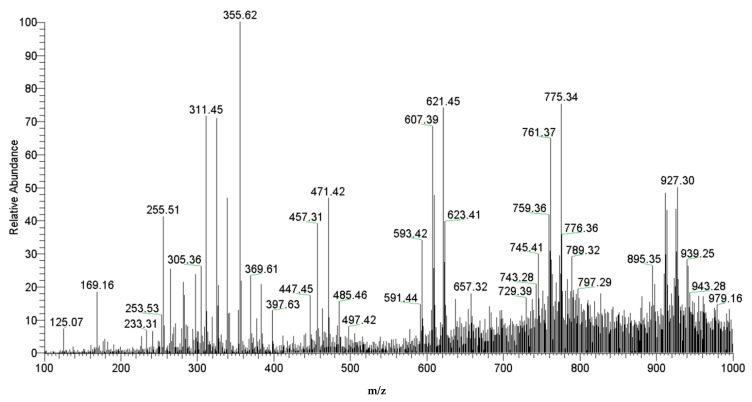
Representative full-scan PS-MS profile of *Stryphnodendron adstringens* (Mart.) Coville extract in negative ionization mode.

**Figure 8:**
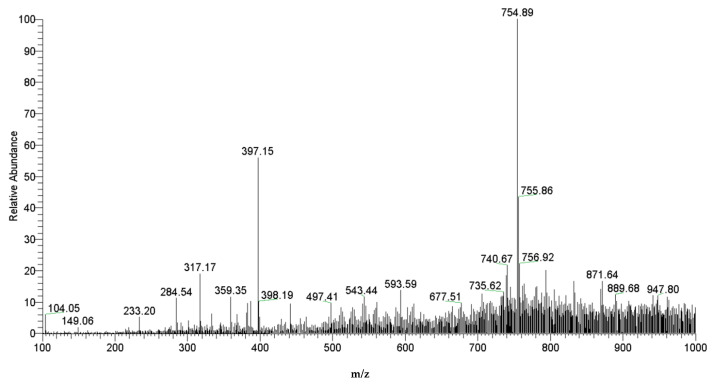
Representative full-scan PS-MS profile of *Baccharis crispa Spreng* extract in positive ionization mode.

**Figure 9:**
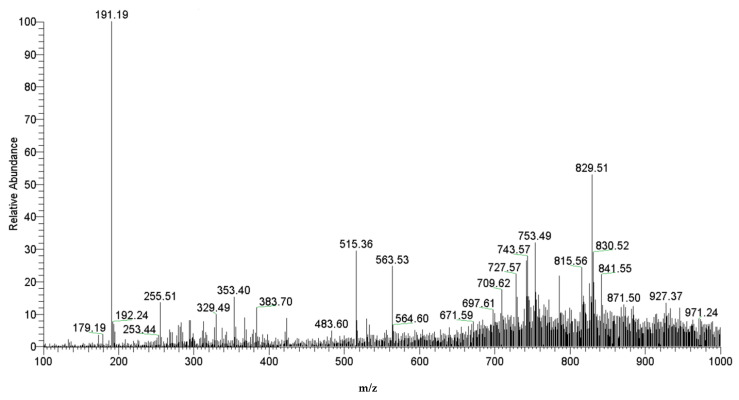
Representative full-scan PS-MS profile of *Baccharis crispa Spreng* extract in negative ionization mode.

**Figure 10:**
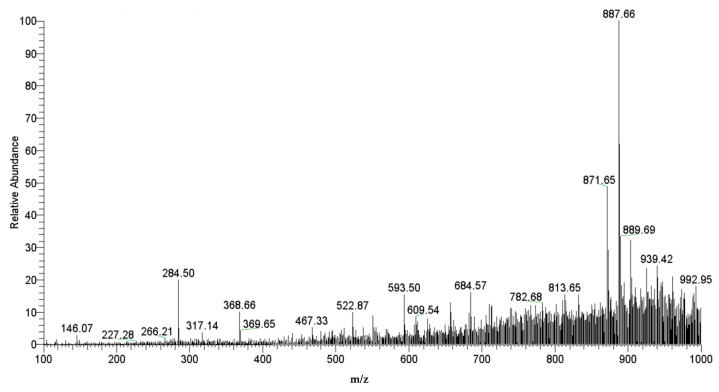
Representative full-scan PS-MS profile of *Azadirachta indica* oil in positive ionization mode.

**Figure 11:**
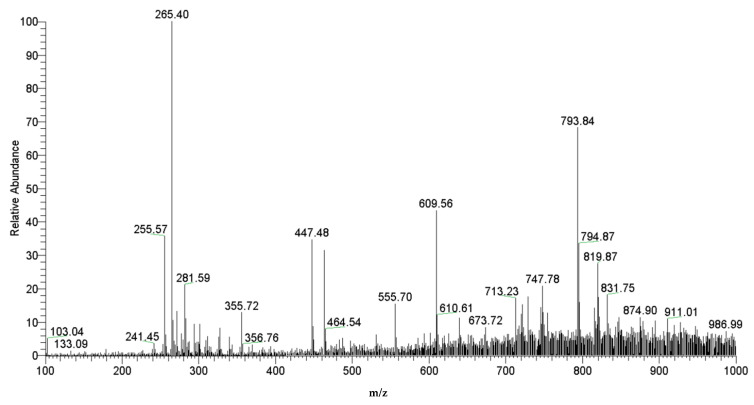
Representative full-scan PS-MS profile of *Azadirachta indica* oil in negative ionization mode.

The PS-MS profiles provide a descriptive chemical context for the antimicrobial results because they show that the evaluated preparations should be considered chemically distinct matrices rather than interchangeable plant extracts. The stronger and broader bactericidal activity observed for *S. adstringens* extract is consistent with a profile enriched in signals compatible with hydrolysable tannins, flavonoid derivatives, and phenolic constituents, chemical classes frequently associated with protein-binding capacity, cell-envelope interactions, and impaired microbial growth. By contrast, the more restricted activity of *B. crispa* and the differential responses observed between *A. indica* extract and oil suggest that antimicrobial performance is influenced not only by plant species, but also by extraction matrix and compound polarity. Thus, PS-MS contextualizes the MBC data by showing that the evaluated preparations are chemically distinct matrices; however, no association between specific ion signals and the observed bactericidal patterns can be established based on the present data. Definitive causal relationships will require future LC-HRMS, bioactivity-guided isolation, quantification, and compound-specific assays. It should be emphasized that the spectra presented in Figure 6, Figure 7, Figure 8, Figure 9, Figure 10 and Figure 11 correspond to full-scan PS-MS profiles rather than structurally validated MS/MS spectra. Therefore, the reported metabolite assignments are interpreted exclusively as tentative MSI Level 3 annotations supported by nominal mass comparison and limited supportive fragmentation data.

Profiles obtained for *Stryphnodendron adstringens* (Mart.) Coville (ESa), *Baccharis crispa* Spreng (EBc), and *Azadirachta indica* (EAi and OAi) suggested the presence of signals compatible with hydrolysable tannins, flavonoids, proanthocyanidins, phenolic acids, and, in the case of *A. indica* oil, more lipophilic constituents. Under the analytical conditions employed, these assignments are interpreted as tentative annotations based on nominal mass comparison with literature data, with supportive MS/MS information for selected ions. The spectra presented in this work are full-scan PS-MS profiles and are not annotated MS/MS spectra. The MS/MS data acquired during the analysis were not sufficiently diagnostic for structural confirmation under the instrumental conditions employed and were therefore used only as exploratory supportive information, consistent with the MSI Level 3 annotation level adopted throughout this study. PS-MS results are presented in this study as an exploratory qualitative overview of broad chemical classes present in the extracts and oil rather than as definitive compound identification. Although such classes have been reported in the literature as relevant to antimicrobial activity, further continued assays employing complementary methodology are required to establish a definite causal relationship between individual metabolites and the bactericidal effects observed for the crude extracts and oil.

#### 3.2.1. *Stryphnodendron adstringens* (Mart.) Coville

Fourteen ions in negative ionization mode and eight in positive ionization mode were tentatively annotated for *Stryphnodendron adstringens* (Mart.) Coville. Detected ions suggest a phytochemical profile particularly composed of polyphenolic and flavonoid derivatives, which are commonly associated with antioxidant and anti-inflammatory activities. These findings are consistent with the ethnopharmacological literature on *Stryphnodendron adstringens* (Mart.) Coville [35,36]. The tentative annotation of signals compatible with hydrolyzable tannins, flavonoid glycosides, and phenolic acids provides a descriptive overview of the chemical classes present in the extract under the analytical conditions employed; however, no functional or biological relevance can be attributed to these annotations based on the present data [37,38].

Related to PS-MS analysis, in the positive ionization mode (Figure 6), an ion tentatively annotated as ellagic acid (*m*/*z* 303) was observed; this polyphenol has been reported in the literature in relation to antioxidant properties [39], though no direct assessment of this activity was performed in the present study. An ion tentatively annotated as HHDP-digalloylglucoside, also known as tellimagrandin I (*m*/*z* 787), was observed, with limited fragmentation support. This compound class has been discussed in the literature in relation to antiviral and antimicrobial properties [40]; however, no compound-specific biological testing was conducted here, and no causal relationship is implied. An ion tentatively annotated as Riparin III (*m*/*z* 288) was observed, an alkaloid previously reported in other plant species; however, its biological relevance in the context of antimicrobial activity was not directly assessed in this study [41]. An ion tentatively annotated as casuariin (*m*/*z* 785), a hydrolyzable tannin, was observed; this chemical class has been discussed in the literature in relation to astringent and antimicrobial properties [42], though no compound-specific validation was performed in this study.

The negative ionization mode (Figure 7) tentatively annotated several flavonoid and phenolic glycoside-related ion signals, suggesting the presence of diverse polyphenolic and flavonoid-related chemical classes of *Stryphnodendron adstringens* (Mart.) Coville. The following discussion presents tentatively assigned identifications for each signal at their respective *m*/*z* in the context of each species. Additional ions were tentatively annotated as caffeic acid-hexoside (*m*/*z* 341), rutin (*m*/*z* 609), punicalin (*m*/*z* 781), quercetin-3,4′-O-diglucoside (*m*/*z* 625), and cyanidin-O-glucosyl-O-acetylpentoside (*m*/*z* 623). These annotations are based on nominal *m*/*z* comparison with literature data and are interpreted as MSI Level 3 putative assignments. The corresponding chemical classes—phenolic acids, flavonoid glycosides, ellagitannins, and anthocyanin derivatives—have been reported in the literature in relation to various biological activities [43,44,45,46]; however, none of these activities were directly assessed in the present study, and no causal relationship between these signals and the antimicrobial outcomes is implied. ESa exhibited the lowest MBCs against Gram-positive bacteria and retained antimicrobial activity, at higher concentrations, against *E. coli* and *Salmonella* spp. PS-MS analysis suggested the presence of multiple signals compatible with ellagitannins, gallotannins, flavonoid glycosides, and phenolic acid derivatives. These chemical classes have been reported in the literature as potentially relevant to antimicrobial activity, particularly in tannin-rich plant matrices.

#### 3.2.2. *Baccharis crispa* Spreng

For *Baccharis crispa* Spreng, eight ions in negative ionization mode and five in positive ionization mode were tentatively annotated, majorly composed of hydrolyzable tannins, flavonoids, and phenolic acids. This tentative phytochemical profile is broadly consistent with the chemical classes previously reported for species used in traditional hepatoprotective and anti-inflammatory contexts [47,48]. These annotations are presented as descriptive context only and do not imply functional validation or direct applicability in the present study [49,50].

Among the positively ionized compounds tentatively identified (Figure 8), an ion annotated as casuariin (*m*/*z* 785) was observed, a tannin class previously associated in the literature with antimicrobial-related and antioxidant activities. An ion tentatively annotated as octadecanoic acid (*m*/*z* 285) was also observed, a saturated fatty acid; this chemical class has been discussed in the literature in relation to various biological properties [42,51], though no direct assessment was performed here. An ion tentatively annotated as Tellimagrandin I (*m*/*z* 787) was also observed. This hydrolyzable tannin class has been reported in the literature in relation to antimicrobial properties [40,52], though no compound-specific biological testing was conducted in the present study.

The negative ionization mode (Figure 9) tentatively observed ferulic acid (*m*/*z* 193), previously discussed in the literature in relation to antioxidant and anti-inflammatory properties. Additional ions were tentatively annotated as HHDP-digalloylglucose (*m*/*z* 785), procyanidin trimer A (*m*/*z* 863), and galloyl-bis-HHDP-glucose (*m*/*z* 935). The chemical classes corresponding to these signals have been discussed in the literature in relation to antioxidant and hepatoprotective properties [53,54,55,56,57]; however, these are literature-based observations and were not directly evaluated in the present study. EBc contained proanthocyanidins, HHDP tannins, ferulic acid, and flavonoids, and exhibited antimicrobial activity predominantly against Gram-positive bacteria, with no detectable MBC against *E. coli* or *Salmonella* spp. at concentrations up to 100 mg/mL. PS-MS profiling suggested the presence of signals compatible with proanthocyanidins, hydrolysable tannins, phenolic acids, and flavonoid-related compounds. These chemical classes have been reported in the literature in relation to antimicrobial and anti-adhesion effects, but the present study did not include quantitative analysis, compound-specific testing, or mechanistic validation. Accordingly, these phytochemical observations should be interpreted only as descriptive context for the crude extract.

#### 3.2.3. *Azadirachta indica*

The PS-MS analysis of *Azadirachta indica* yielded six tentatively annotated ions in negative ionization mode and three in positive ionization mode. Signals tentatively annotated in this matrix are broadly consistent with chemical classes reported in the literature for neem preparations [58,59,60]; however, these annotations do not constitute evidence of antimicrobial, anti-inflammatory, or neuroprotective activity in the present study.

In the positive ionization mode (Figure 10), an ion tentatively annotated as octadecanoic acid (*m*/*z* 285) was observed; this fatty acid class has been discussed in the literature in relation to dermatological and lipid metabolism contexts [51,61], though no direct assessment was performed here. An ion tentatively annotated as Tellimagrandin I (*m*/*z* 787) was also observed, with literature citing this compound as possessing antimicrobial properties [40,52].

The negative ionization mode (Figure 11) tentatively observed additional ions: they were annotated as dihydroquercetin-3,5-rhamnoside (*m*/*z* 449), delphinidin-3-O-glucoside (*m*/*z* 465), procyanidin trimer A (*m*/*z* 863), and galloyl-bis-HHDP-glucose (*m*/*z* 935). The chemical classes corresponding to these signals (i.e., flavonoids, anthocyanins, and condensed and hydrolysable tannins) have been reported in the literature in relation to antioxidant, anti-inflammatory, and antimicrobial properties [62,63,64,65]. These are presented here strictly as literature-based contextual observations; no compound-specific biological activity was assessed in the present study and no causal attribution to the observed antimicrobial outcomes is implied.

Several recurring ion signals at the same nominal *m*/*z* values were tentatively annotated across multiple plant matrices (Table 2), including signals compatible with tellimagrandin I (*m*/*z* 787), casuariin (*m*/*z* 785), procyanidin trimer A (*m*/*z* 863), and galloyl-bis-HHDP-glucose (m/z 935). The recurrence of these ion signals across multiple matrices is noted as a descriptive phytochemical observation only and does not imply that these chemical classes are responsible for the antimicrobial activity observed in the present study. OAi contained flavonoids, anthocyanins, and tannins, and achieved minimum bactericidal concentrations (MBCs) against the evaluated Gram-negative bacteria, whereas EAi did not reach MBC values against *Escherichia coli* at concentrations (100 mg/mL). The extracts exhibited signals tentatively associated with constituents related to flavonoids, anthocyanins, and tannins, while the oily matrix displayed a distinct profile with more lipophilic characteristics. Differences between the extract and oil matrices may help contextualize the distinct antimicrobial responses observed, particularly against Gram-negative bacteria.

#### 3.2.4. Recurring Ion Signals and Comparative Phytochemical Interpretation

Several recurring ion signals were tentatively annotated across more than one plant matrix at the same nominal *m*/*z* values, including signals at *m*/*z* 787, 785, 863, and 935, tentatively corresponding to tellimagrandin I, casuariin, procyanidin trimer A, and galloyl-bis-HHDP-glucose respectively, under the analytical conditions employed. The recurrence of these ion signals across chemically distinct preparations—an ethanolic bark extract, an aqueous aerial-part decoction, an ethanolic leaf extract, and a cold-pressed seed oil—suggests that these chemical classes represent a broadly distributed phytochemical baseline among the evaluated species rather than species-specific markers. Despite this overlap, the differential antimicrobial performance observed (ESa > OAi ≈ EAi > EBc) indicates that biological activity is shaped not by the mere presence of shared compound classes but by their relative abundance, the polarity of the extraction matrix, and the overall compositional context of each preparation. These findings may support the interpretation that the extracts should be treated as whole matrices rather than as equivalent vehicles for the same set of bioactive molecules.

#### 3.2.5. Mechanism-Outcome Integration and Implications

The comparative bactericidal response pattern observed among the evaluated matrices and the greater susceptibility of Gram-positive bacteria relative to Gram-negative strains may be tentatively discussed in relation to differences in the chemical classes tentatively annotated across the evaluated matrices; however, no mechanistic attribution is supported by the present data, and this interpretation should be regarded as hypothesis-generating only. Although PS-MS enables rapid qualitative fingerprinting with minimal sample preparation, the present data do not allow attribution of bactericidal effects to discrete molecules or compound combinations. Translational advancement will require bioactivity-guided fractionation, LC-HRMS quantification, and targeted mechanistic assays (e.g., membrane integrity, enzymatic inhibition, time–kill, and biofilm assays) before any compound-level or mechanism-level conclusions can be drawn.

The results provide a preliminary basis for further pharmacological and clinical investigations [40,42,52,55,56,57]. This concentration-resolved visualization complements the MBC analysis by illustrating how extracts and oil matrix and bacterial cell envelope structure jointly shape bactericidal outcomes, reinforcing the integrative framework proposed in this study. However, one important aspect that should be emphasized is that the findings are based on crude extracts and oil. Therefore, additional refinement, formulation strategies, or processing approaches, as well as future investigations employing alternative extraction solvents (e.g., alcohol-based solvents), may contribute to the identification of distinct bioactive profiles and to distinct assessments of their potential benefits, as exemplified before.

Although the MBC values recorded for most extracts (i.e., EBc, EAi, and Oai) fall in the range of 50–100 mg/mL, which exceeds concentrations typically achievable in systemic therapies, these values are not necessarily prohibitive in the context of topical or preventive applications. Pre- and post-milking teat dipping allows for direct surface-level application of concentrated plant-derived formulations without the pharmacokinetic constraints associated with oral or parenteral routes. In this scenario, the bactericidal concentrations identified here may be technically achievable in a formulated dipping solution, particularly for pathogens such as *S. aureus* and *S. uberis*, which exhibited the highest susceptibility across extracts. Translational development of such formulations will, however, require evaluation of extract stability, skin compatibility, and efficacy under field conditions.

An additional limitation of the present study is that the extracts and oil were not subjected to detailed phytochemical standardization based on quantitative marker compounds or validated compositional specifications. Likewise, the PS-MS workflow employed here was not designed to provide definitive structural characterization of individual constituents in these complex matrices. Therefore, although phytochemical screening offers a useful exploratory overview of broad chemical classes present in the samples, it does not allow identification of the specific compounds responsible for the observed antimicrobial activity. For this reason, the antimicrobial findings should be interpreted at the level of the crude extracts and oil as whole matrices under the tested conditions.

## 4. Conclusions

Under the experimental conditions evaluated, the extracts exhibited bactericidal activity against selected bacteria isolated from bovine mastitis. Among them, the extract of *S. adstringens* (Mart.) Coville showed comparatively lower MBC values across the tested strains, indicating a relatively higher activity under the conditions evaluated. The *S. uberis* strain displayed lower resistance in comparison to the other bacteria, regardless of the extract or oil assessed. Although the activity of *S. adstringens* (Mart.) Coville was more pronounced, the extracts and oil demonstrated a limited but detectable antimicrobial effect.

It should also be noted that the bacterial panel employed in this study, comprising five strains representative of the most prevalent contagious and environmental mastitis pathogens, does not capture the full diversity of microorganisms associated with bovine mastitis, and the findings should not be extrapolated to species or strains not evaluated here. Furthermore, the present study did not assess cytotoxicity, tissue irritation, or animal safety, which are essential parameters for any future consideration of veterinary application and must be addressed in subsequent investigations before translational use can be contemplated.

Paper Spray Mass Spectrometry was employed as a complementary exploration tool for the tentative annotation of broad chemical classes present in the evaluated matrices. The phytochemical profiles obtained provide descriptive context for the antimicrobial findings and may inform the design of future targeted investigations, but do not constitute mechanistic or functional evidence. The combination of MBC assays and GLM outcome modeling with qualitative PS-MS fingerprinting represents an exploratory integrative approach, the scope of which is strictly limited to hypothesis generation under the analytical conditions employed. The differential bactericidal responses observed between extracts and neem oil further highlight the influence of extraction matrix and compound polarity on antimicrobial performance. This matrix-dependent effect, particularly evident in the activity against Gram-negative bacteria, underscores that antimicrobial potential cannot be attributed solely to plant species but is strongly conditioned by physicochemical properties of the extract. Therefore, further studies are warranted to better elucidate the role of these compounds through bioactivity-guided fractionation and targeted metabolomic approaches. The exploratory integrative framework applied here may serve as a preliminary basis for prioritizing plant-derived matrices in future investigations, provided that the limitations of the qualitative PS-MS approach and the absence of compound-level validation are considered. Further studies employing bioactivity-guided fractionation, high-resolution MS, quantitative analysis, and in vivo models will be necessary before any translational or clinical conclusions can be drawn.

## 5. Patents

In the context of intellectual property protection, the invention patent “Herbal Solution for Pre- and Post-Dipping” also known as “Natural Dipping” has been filed under the Brazilian National Application for invention, utility model, certificate of addition of invention, and entry into the national phase of the Patent Cooperation Treaty (PCT). The patent application, registered under Process BR 10 2021 009874 0, was officially submitted on 21 May 2021.

## Figures and Tables

**Table 1:** Minimum bactericidal concentration (MBC) of ESa, EBc, EAi, and OAi against strains of *S. aureus*, *S. agalactiae*, *S. uberis*, *E. coli*, and *Salmonella* spp. isolated from cases of bovine mastitis.

Bacterium	Concentration of Extracts and Oil (mg/mL)			
	ESa	EBc	EAi	OAi
*S. aureus*	1.56	100	25	100
*S. agalactiae*	3.12	100	12.5	100
*S. uberis*	1.56	50	6.25	50
*E. coli*	50	>100 *	>100 *	100
*Salmonella* spp.	6.25	>100 *	100	100

* Showed bacterial growth in all concentrations. ESa = Extract of *Strypnodendron adstringens* (Mart.) Coville; EBc = Extract of *Baccharis crispa* Spreng; EAi = Extract of *Azadirachta indica*; OAi = Oil of *Azadirachta indica*. Concentrations do not differ between columns but differ between rows (*p* < 0.001).

**Table 2:** General overview of major ion signals and their tentatively annotated chemical classes by PS-MS (putative annotations, MSI Level 3) in the evaluated plant species, their literature-reported biological association and the level of evidence in this study. Previously reported biological associations (literature-based).

Plant Species	Major Ions Tentatively Annotated (PS-MS, MSI Level 3)	Chemical Class	Previously Reported Biological Associations (Literature-Based)	Evidence Level in the Present Study *
*Stryphnodendron adstringens* (Mart.) Coville	Tellimagrandin I (*m*/*z* 787)	Hydrolyzable tannin	Reported bacteriostatic and bactericidal effects, mainly against Gram-positive bacteria; associated with protein precipitation and cell wall interactions	Antimicrobial activity demonstrated only at crude extract level
	Casuariin (*m*/*z* 785)	Hydrolyzable tannin	Associated with antimicrobial and astringent activity; may affect membrane permeability	Antimicrobial activity inferred from literature
	Punicalin (*m*/*z* 781)	Ellagitannin	Reported antimicrobial and enzyme-inhibitory activity	No direct compound-specific testing
	Rutin (*m*/*z* 609)	Flavonoid glycoside	Reported mild antimicrobial activity and potential synergistic effects	Antimicrobial effect not individually assessed
	Quercetin derivatives (*m*/*z* 625)	Flavonoids	Associated with growth inhibition of Gram-positive bacteria	Activity inferred from literature
	Caffeic acid derivatives (*m*/*z* 341)	Phenolic acids	Reported antimicrobial and oxidative stress-related effects	No direct mechanistic assessment
*Baccharis crispa* Spreng	Casuariin (*m*/*z* 785)	Hydrolyzable tannin	Reported antimicrobial activity, particularly against Gram-positive bacteria	Antimicrobial activity observed at crude extract level
	Tellimagrandin I (*m*/*z* 787)	Hydrolyzable tannin	Associated with antibacterial and protein-binding effects	Compound-specific activity not evaluated
	Ferulic acid (*m*/*z* 193)	Phenolic acid	Reported moderate antimicrobial and membrane-disruptive effects	Antimicrobial relevance inferred
	Procyanidin trimer A (*m*/*z* 863)	Condensed tannin	Associated with reduced bacterial adhesion and biofilm formation	Activity inferred from literature
*Azadirachta indica*	Tellimagrandin I (*m*/*z* 787)	Hydrolyzable tannin	Reported antibacterial activity against Gram-positive bacteria	Antimicrobial activity demonstrated at extract/oil level
	Dihydroquercetin-rhamnoside (*m*/*z* 449)	Flavonoid	Associated with antimicrobial and antioxidant properties	No direct antimicrobial testing
	Delphinidin-3-O-glucoside (*m*/*z* 465)	Anthocyanin	Reported mild antimicrobial effects via membrane interaction	Activity inferred from literature
	Procyanidin trimer A (*m*/*z* 863)	Condensed tannin	Associated with antimicrobial and anti-adhesion effects	No compound-specific validation
	Octadecanoic acid (*m*/*z* 285)	Fatty acid	Reported antimicrobial effects via membrane destabilization, particularly in oil matrices	Antimicrobial activity demonstrated at oil level

* Crude extract/oil level indicates that antimicrobial activity was experimentally demonstrated for the whole extract or oil, without isolation or testing of individual compounds. Literature inference indicates that antimicrobial-related activity is supported by previously published studies for the compound or chemical class but was not directly evaluated in the present work. No compound-specific validation indicates that the compound was tentatively identified by PS–MS and discussed based on chemical class behavior, without direct biological testing.

## Data Availability

The original contributions presented in this study are included in the article. Further inquiries can be directed to the corresponding authors.
